# Comparing Effects of Aromatherapy with Five Herbs Essential Oils on PCPA-induced Insomnia Mice

**DOI:** 10.4014/jmb.2409.09021

**Published:** 2024-11-29

**Authors:** Peipei Feng, Jingyi Chen, Xiaolu Chen, Minghui Tang, Ni Song, Lanyue Zhang, Tinggang He

**Affiliations:** 1 Hua An Tang Biotech Group Co., Ltd., Guangzhou 510000, P.R. China; 2 Guangdong He Ji Biotech Co., Ltd., Guangzhou 510000, P.R. China; 3 School of Biomedical and Pharmaceutical Sciences, Guangdong University of Technology, Guangzhou 510006, P.R. China; 4 Guangdong Provincial Key Laboratory of Plant Resources Biorefinery, School of Biomedical and Pharmaceutical Sciences, Guangdong University of Technology, Guangzhou, 510006, P.R. China

**Keywords:** Essential oils, PCPA, 5-HT_1A_, GABA_A_Rα1, GC-MS

## Abstract

Delayed treatment of insomnia-related symptoms can harm physical health and increase the psychological burden. In addition to oral medications and some physical therapies, aromatherapy can help overcome some treatment-related side effects. Herein, parachlorophenylalanine (PCPA)-induced insomnia was established in Kunming (KM) mice, which were subjected to aromatherapy using five plants (*Jasminum sambac*, *Magnolia denudata*, *Rosa rugosa*, *Aloysia citriodora*, and *Abies balsamea*) essential oils (EOs). To determine the sleep-inducing effect of the five EOs, the rate of change in body weight, sleep latency, and total sleep time in mice were measured. Specific serum indices were used to evaluate the therapeutic effects of the tested drugs and PCPA modeling. Gas chromatography-mass spectrometry (GC-MS) was employed to identify active components in EOs. The five EOs contained multiple identical constituents and were rich in terpenoids, such as α-farnesene (28.42%), linalool (68.84%), and citronellol (23.78%). The EOs exhibiting varying effects on insomnia-induced weight loss. Nissl staining was used to examine and the number of neurons was elevated in the EO-treated groups when compared with the PCPA-induced group; however, the neuronal number was reduced in the hypothalamic tissues of the *R. rugosa* EO (RREO)-treated group. All EOs upregulated the expression of 5-HT1A and GABA_A_Rα1, as demonstrated by immunohistochemistry, western blotting, and reverse transcription-quantitative PCR results. In addition, EOs of *A. citriodora* and *A. balsamea* significantly upregulated the expression of 5HT_1A_ protein, whereas EOs of *J. sambac* and *M. denudata* exerted significantly different effects when compared with the model group, as determined by western blotting.

## Introduction

Insomnia is characterized by difficulty in sleep initiation, duration, consolidation, or quality, resulting in some form of daytime disorder despite ample sleep. Numerous epidemiological studies have estimated that approximately one-third of the adult population worldwide suffers from insomnia or chronic insomnia symptoms [1]. Therapeutic agents to relieve insomnia include benzodiazepine receptor agonists, non-benzodiazepine receptor agonists, selective melatonin receptor agonists, and sedative antidepressants [2]. Most drugs to treat insomnia target serotonin (5-HT) and gamma-aminobutyric acid (GABA) receptors [3]. However, the use of these drugs is accompanied by side effects, such as hangover, psychomotor disorders, drug addiction, tolerance, amnesia, and rebound insomnia, and their clinical effectiveness remains controversial [4]. Therefore, there is an unmet need to identify and develop novel hypnotics with fewer side effects and better efficacy.

Aromatherapy involves the use of essential oils (EOs) derived from various plant parts, have been extensively studied for their therapeutic potential, including their effects on sleep and mood regulation. In traditional medicine, aromatherapy is used to treat chronic pain, depression, anxiety, insomnia, stress, and other mental disorders. Accumulated evidence has suggested that the primary mechanism of aromatherapy may involve the limbic system [5]. Aromatic components stimulate olfactory cells, transmit signals to the brain, and affect the autonomic nervous system and hormone secretion. EOs can directly affect breathing, circulation, and the central nervous system via the skin and airways. The chemical composition of various EOs has indicated their calming and hypnotic effects; however, systematic studies examining these effects are lacking. Since aromatherapy can improve sleep quality [6], the clinical application of EOs in aromatherapy has received growing attention, and detailed studies on the pharmacological effects of inhaled EOs are warranted. The primary objective of the present study was to determine the hypnotic effects of aromatherapy with five specific EOs, and to investigate the association between insomnia and the expression of related proteins in brain tissues.

As a well-established model for inducing serotonin depletion and sleep disturbances, PCPA reduces 5-HT levels by inhibiting tryptophan hydroxylase, mimicking insomnia symptoms. Studies have shown that PCPA-treated mice exhibit behavioral changes similar to chronic insomnia in humans, such as altered sleep cycles and persistent wakefulness [7]. In addition, the PCPA-induced insomnia model was also used to assess the effects of drugs on transient and stress-related insomnia. Studies have shown that PCPA treatment not only affects the 5-HT system, but may also affect dopamine (DA) and norepinephrine (NE) levels [8]. In a PCPA-induced insomnia mouse model, 5-HT secretion in the hypothalamus was significantly inhibited, while NE and DA levels were increased. This suggests that PCPA may induce insomnia by affecting multiple neurotransmitter systems, echoing the complexity of insomnia in humans.

*Jasminum sambac*, belonging to the Oleaceae family, is an important crop widely cultivated in China, India, Egypt, and Morocco [9]. The aroma of *J. sambac* essential oil is believed to improve sleep quality, especially the subjective and objective sleep quality [10]. *Magnolia denudata* belongs to *Magnoliaceae* and is used in traditional medicine to treat colds and headaches in Asian countries [11, 12]. While there is less research directly on the effects of *M. denudata* essential oil on sleep, *M. denudata* essential oil has traditionally been used to relieve anxiety and stress, which may indirectly improve sleep quality [13]. More research is needed to explore its direct effects on sleep. *Rosa rugosa* is a rose species native to eastern Asia and grows on beach coasts [14]. Previous trials have explored the therapeutic efficacy of rose EOs, including their antidepressant effects and psychological relaxation potential [15]. *Aloysia citriodora* is a plant native to South America and recent study has revealed that *A. citriodora* is a natural source of functional ingredients for treating several disorders [16, 17]. *Abies balsamea* is a conifer native to northeastern North America [18] and is known to possess multiple medicinal uses. Also, *A. citriodora* and *A. balsamea* has the potential to improve sleep quality and severity of insomnia in patients with insomnia [19, 20]. The EOs from these plants are rich in terpenoids with diverse biological activities. For example, linalool in *J. sambac* and *M. denudata* has sedative effects by modulating GABA_A_ receptors [21]. The α-Farnesene in *R. rugosa* has neuroprotective properties relevant to sleep regulation [22]. *A. citriodora*, high in citral, has anti-inflammatory and antioxidant activities beneficial for insomnia [16]. *A. balsamea*, containing bornyl acetate, has anti-depressant effects, supporting its role in insomnia management [20].

Mass spectrometry is an analytical method with high sensitivity and a powerful tool for the identification of the structure of active components. Gas chromatography-mass spectrometry (GC-MS) technology combined with chromatography has the advantages of wide application range, high selectivity, low detection limit, high efficiency, and fast analysis speed. In the present study, we compared the chemical analysis and aromatherapy mechanisms of five herbal EOs in a parachlorophenylalanine (PCPA)-induced insomnia mouse model. GC-MS was performed to analyze the EOs components and explore its effective sedative and sleeping components.Nissl staining was used to document changes in the number of neurons in brain tissues of different treatment groups. Expression levels of 5-HT_1A_ and GABA_A_Rα1 in mouse brain tissues were determined by immunohistochemistry, western blotting, and reverse transcription-quantitative PCR (RT-qPCR). Moreover, studies are ongoing to uncover the relationship between these EOs for treating insomnia disorders. Using a PCPA-induced insomnia model, we investigated the effects of the EOs on sleep latency, sleep duration, and neuronal protein expression. Our results suggest that certain EOs may have hypnotic effects and may be useful in the management of insomnia. By examining the hypnotic effects of these EOs and their impact on neuronal proteins involved in sleep regulation, our study aims to provide novel insights into the potential therapeutic use of aromatherapy for insomnia.

## Methods and Materials

### Material and Preparation of EOs

Diazepam (DZP) was acquired from Guangzhou Xinshi Hospital. PCPA was of analytical grade and purchased from Aladdin Reagent Database Inc. (China). *J. sambac*, *M. denudata*, *R. rugosa*, *A. citriodora*, and *A. balsamea* were authenticated by Professor Nian Liu from Zhongkai University of Agriculture and Engineering (China). Table 1 presents information regarding the plant collection and extraction procedures.

The fresh plants were washed and after drying at 40°C for 72 h, pulverize and sieve, take 40 mesh powder for use. 500 g powder was mixed with 4 L distilled water, packed into a cloth bag, tied tightly, added 20 g sodium chloride, and distilled in high efficiency essential oil distillation equipment (TX05-02, China). Distillation at 2200 W until reflux occurs, then 800 W for 3.5 h. Then, EOs were separated from the oil-water mixture. Each EO was dried over anhydrous sodium sulfate and stored at 4°C.

### GC-MS Analysis of EOs

EOs was analyzed using a GCMS-QP2010 PLUS (Shimadzu Co., Japan) equipped with a 30 mm DB-5 ms capillary column. Helium was used as the carrier gas at a flow rate of 0.87 ml/min. The chromatographic temperature was programmed as follows: the initial temperature was set at 90°C for 10 min, heated to 250°C at a rate of 5°C/min, and maintained at 250°C for 8 min. The temperature of the gasification chamber was 250°C with a shunt ratio of 100:1. The mass spectrometry conditions were as follows: electron energy, 70 eV; ion source temperature, 200°C. The retention index (RI) of each compound was calculated following the n-alkane standard (C_7_–C_40_).

Compounds containing EOs were confirmed by comparing their RI and mass spectra with those of standard compounds available on NIST MS Library. The components of the compounds were identified by comparison with the NIST mass spectrometry library data and the essential oil compounds obtained by GC-MS, and only the components with a small difference from the reference value RI were retained.

### Animal Grouping

The experimental protocol was approved by the Experimental Animal Center of Guangdong Province (approval documents: SCXK/20130002). Male, 5-weeks-old Kunming (KM) mice weighing 25 g were randomly divided into eight groups, with 10 mice in each group: EOs, PCPA, DZP, and control groups.

### Establishment of the PCPA-induced Insomnia Model and Treatment

PCPA can shorten sleep duration by blocking the synthesis of 5-HT, and absolute insomnia can be induced by treatment with PCPA 24 h before pentobarbital injection. In the five EOs groups, mice sniffed EO-soaked filter paper placed in four corners of the cage for 1 h [23], followed by intraperitoneal PCPA dissolved with saline containing 1% Tween 80 (30 mg/ml) on days 4 and 5. Animals in the PCPA and DZP groups sniffed saline filter paper followed by intraperitoneal PCPA dissolved with saline containing 1% Tween 80 (30 mg/ml) on days 4 and 5. The PCPA suspension was intraperitoneally injected into mice at a predetermined time point daily. The control group was injected with the same volume of weakly alkaline normal saline (1% Tween 80, pH 7-8) in the same manner. The body weight of mice was recorded daily prior to EO sniffing and drug administration. After the last intraperitoneal injection (26–30 h), behavioral changes were observed. Compared with healthy controls, all PCPA-treated mice exhibited a disrupted circadian rhythm and increased water and food consumption. Mice also become irritable and excitable. Moreover, evident sexual behaviors, such as sleep time, were significantly reduced. These changes indicated successful model establishment.

### Pentobarbital-Induced Sleep Test

To evaluate the hypnotic effects of EOs, a pentobarbital-induced sleep test was performed as previously described. Animals inhaled EOs or solvents for 1 h or 30 min after intraperitoneal DZP dissolved with saline containing 1% Tween 80 (0.2 mg/ml) and were subsequently administered pentobarbital sodium (1%, 45 mg/kg) to induce sleep for behavioral testing [24]. Hypnotic drugs typically prolong the pentobarbital sodium-induced sleep time. During experimentation, each mouse sniffed the respective EO for 60 min and was administered a threshold dose of sodium pentobarbital intraperitoneally to record the time to fall asleep and sleep time, as well as draw a curve of EO concentration accordingly. Sleep latency was defined as the time between pentobarbital injection and falling asleep, and sleep duration was defined as the time difference between the loss and recovery of righting reflex.

### Nissl Staining

On day 7, mice in each group were anesthetized with pentobarbital sodium (1%, 50 mg/kg), perfused with 0.1 M phosphate-buffered saline (PBS; pH 7.4) for 10 min, and then treated with 4% paraformaldehyde in 0.1 M fixed in phosphate-buffered saline (PB, pH 7.4) for 10 min.The brains were removed and soaked in 4% paraformaldehyde for 48 h. Paraffin-coated sections of the cortex, hippocampus, and hypothalamus (4-μm thick) were stained with 1% toluidine blue solution (Beyotime, China) for approximately 30 min, washed with double-distilled water, and the colors were separated with 2% hydrochloric acid ethanol. Next, the sections were rinsed with distilled water, dehydrated with graded ethanol, and coated with a neutral resin. The prepared paraffin sections were placed on a slide scanner for imaging, and ImagePro 6.0 was used to count normal nerve cells.

### Immunohistochemistry

Tissue sections were sequentially deparaffinized in xylene, absolute ethanol, and ethanol at different concentrations. After slicing, the antigens were extracted by microwave heating in citric acid (pH 6.0) antigen extraction buffer for 30 min and then blocked in 3% hydrogen peroxide. Subsequently, 3% bovine serum albumin (BSA) was added dropwise to block the tissue for 30 min; the diluted primary antibody (5HT_1A_, GABA_A_Rα1) was added and incubated overnight at 4°C. The following day, secondary antibodies were added, and DAB and hematoxylin staining was performed. The slides were observed under a microscope, and positive cells were analyzed using ImagePro 6.0.

### Western Blot Analysis

Briefly, harvested hippocampal tissue was dissolved in 0.01 mM phenylmethanesulfonyl fluoride (PMSF) ice-soluble buffer and subsequently crushed in an ice bath using a homogenizer. The samples were centrifuged at 10,000 rpm for 10 min at 4°C. The supernatant was stored at -80°C for further use. We used the BCA protein detection kit to measure the protein concentration in the brain in accordance with the manufacturer’s instructions. Samples were loaded into sodium dodecyl sulfate-polyacrylamide gel electrophoresis (SDS-PAGE) loading buffer and boiled for 5 min for protein denaturation. Before western blotting, 10 and 8% SDS-polyacrylamide gels were prepared. Equal amounts of protein (50 μg) were separated on an SDS-polyacrylamide gel for 2 h and then transferred to polyvinylidene fluoride membranes. The protein membrane was blocked with 5% BSA or 5% skimmed milk at room temperature for 2 h and then incubated with the primary monoclonal antibody at 4°C overnight. Anti-5-HT_1A_ and GABA_A_Rα1 primary antibodies were diluted to 1:1000, and β-actin was diluted to 1:1000. All membranes were washed with Tris-buffered saline containing 0.1% Tween 20 (TBST)(3 × 10 min) and incubated with the secondary antibody for 2 h at room temperature. The samples were then rinsed with TBST 3 times (3 × 10 min). A detection system was employed, and the relative intensity was normalized to that of β-actin (internal standard).

### Cell Culture

BV2 and HA1800 cells were inoculated with 89% DMEM containing 10% FBS and 1% penicillin-streptomycin solution prepared in advance, and incubated in a cell culture incubator (Unity Lab Services, USA) in a constant temperature 37°C and 5% CO_2_ atmosphere and saturated humidity. The nutrient solution was changed every day.

### MTT Assay

When the BV2 and HA1800 cells could be observed under the microscope to cover 80% of the bottom area of the culture flask, the medium was poured out and washed with PBS, 1.5 ml of 0.25% trypsin containing EDTA was added, and digested in a constant temperature incubator at 37°C for 3-5 min. 5 ml of medium was added and mixed, then centrifuged at 1,000 rpm for five minutes, and the supernatant was removed. The cells were resuspended in fresh medium and grown in a ratio of 1:3. After repeated above steps, cell counting was performed, and a cell suspension with a concentration of 30,000 to 50,000 cells /ml was finally obtained.

After the cells grow to a certain number, cells were added to 96-well plates at a density of 1 × 10^5^ cells every well. Futher, 100 ng/ml DMEM was added to each well, then returned to the incubator for an additional 24 h.

After 4 h of culture, 100 μl of MTT was added to each well, and 100 μl of DMSO was added to each well. The absorbance value of the well at 570 nm optical density was measured by a microplate reader, and the IC_50_ value was calculated according to the absorbance change.

### Enzyme-Linked Immunosorbent Assay (ELISA)

BV2 cells were seeded in 12-well plates at 5 × 10^5^ times per well and cultured for 4 h in DMEM containing 10%fetal bovine serum. After inoculation and adherence, DMEM containing 2% fetal bovine serum and LPS, LPS+JSEOs, LPS+MDEOs, LPS+ACEOs, LPS+ABEOs, and LPS+RREOs were added. Cells in the culture medium were used as the control group. The cells were incubated at a constant temperature 37°C and 5% CO_2_ atmosphere for 12 h, and the supernatant was stored at -20°C for inflammatory factor detection according to the kit instructions.

### RNA Isolation and RT-qPCR Analyses

RNA was extracted from primary cells cultured in 4 cm^2^ wells using a three-reagent isolation system (Sigma-Aldrich). Each experimental point was analyzed in triplicate. The yield and integrity of RNA were determined using the A_260_ method and agarose gel electrophoresis, respectively. RT-qPCR was performed using the gene-specific primer set (Table 2) designed by the OLIGO 6 software, and amplicon fragments of comparable size (approximately 100 bp) were obtained. mRNA levels of target genes (5HT_1A_ and GABA_A_Rα1) were normalized to mRNA levels of reference genes and glyceraldehyde-3-phosphate dehydrogenase. Normalized values were used to calculate the ratio of expression levels (relative fold change) in treated samples to untreated control samples, according to the 2^-ΔΔCt^ method.

### Statistical Analysis

Data analyses were performed using GraphPad Prism software 8.0.2 (GraphPad Software, USA). Data were analyzed using one-way analysis of variance, the post-hoc tests method is Dunnett's Test, with *p* < 0.05 indicating a significant difference.

## Results and Discussion

### GC-MS Analysis

Herein, EOs were evaluated using GC-MS. Table 3 presents the volatile aroma compounds identified in EOs by GC-MS analysis. A total of 53 compounds were identified in the five kinds of EOs. Given that these plants originate from different species, they contain several compounds, including linalool, β-Ocimene, caryophyllene oxide, methyleugenol, and δ-Cadinene. The total relative contents of RREOs, MDEOs, JSEOs, ACEOs and ABEOs were 74.61%, 81.82%,73.70%, 74.91% and 92.18% respectively. The major compounds in RREOs were citronellol (23.78%), 3,7-dimethyl-2,6-octadien-1-ol (10.76%), and α-farnesene (7.87%). The key compositions in MDEOs were linalool (68.84%) and α-Caryophyllene (3.29 %), while those in JSEOs were α-Farnesene (28.42%), and linalool (16.52%). The dominate components in ACEOs were linalool (60.27%) and Caryophyllene oxide (4.70%), but those in ABEOs were Acetic acid bornyl ester (38.85%), (+)-Limonene (26.53%) and β-ocimene (16.89%). On the other hand, some components such as caryophyllene oxide were in five EOs.

### Sleep Quality and Body Weight

**Fig. 1:**
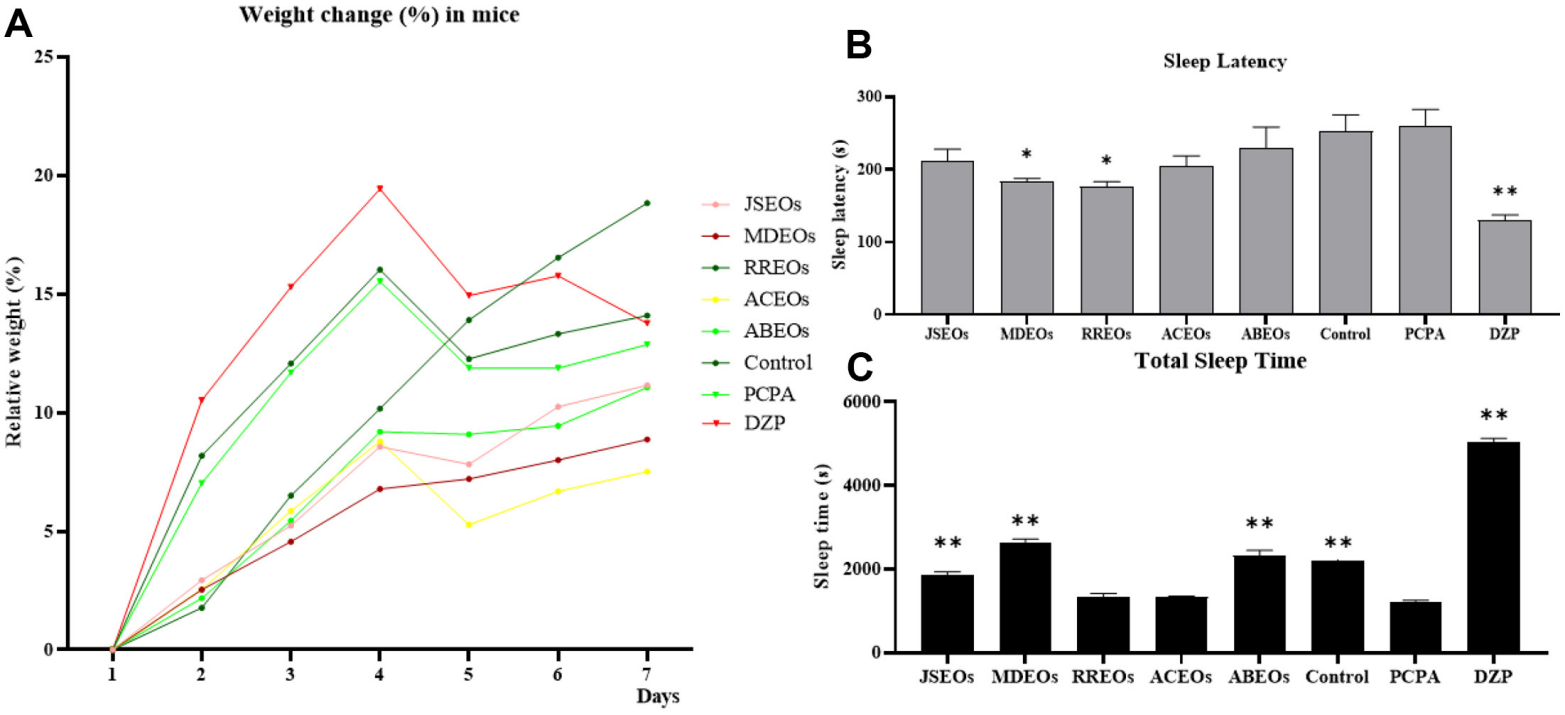
Rate of change in mouse body weight (A) sleep latency (B) and total sleep time (C). Significantly differs from the PCPA group, *p < 0.05 and **p < 0.01. Ordinary one-way ANOVA analysis is used, and the post-hoc test method is Dunnett's Test.

To determine the curative activity of EOs, we evaluated the effect of EOs on the onset and duration of pentobarbital-induced sleep in mice. Variations in daily body weight are shown in Fig. 1A. Fig. 1B and 1C present the effects of EOs on sleep latency and sleep time. Based on a one-sided analysis of variance, after PCPA administration on days 4 and 5, the EO-treated group had less weight loss when compared with the control group. However, the DZP group did not gain significant body weight during this period (Fig. 1A). These findings suggest that EOs reversed the symptoms of weight loss in insomnia mice in a PCPA-induced insomnia model more effectively than a positive control group. The MDEOs-treated and the RREOs-treated groups showed significantly reduced sleep latency when compared with the control group (*p* < 0.05), suggesting that oral these two EOs could effectively induce sedation and hypnosis in the examined behavioral models (Fig. 1B). Notably, while the control group and DZP group significantly increased the total sleep time of mice, JSEOs, MDEOs and ABEOs also played a positive role in prolonging sleep time (*p* < 0.01) (Fig. 1C). Overall, the results showed that EOs afforded significant protection against PCPA-induced insomnia in mice.

### Nissl Staining

**Fig. 2:**
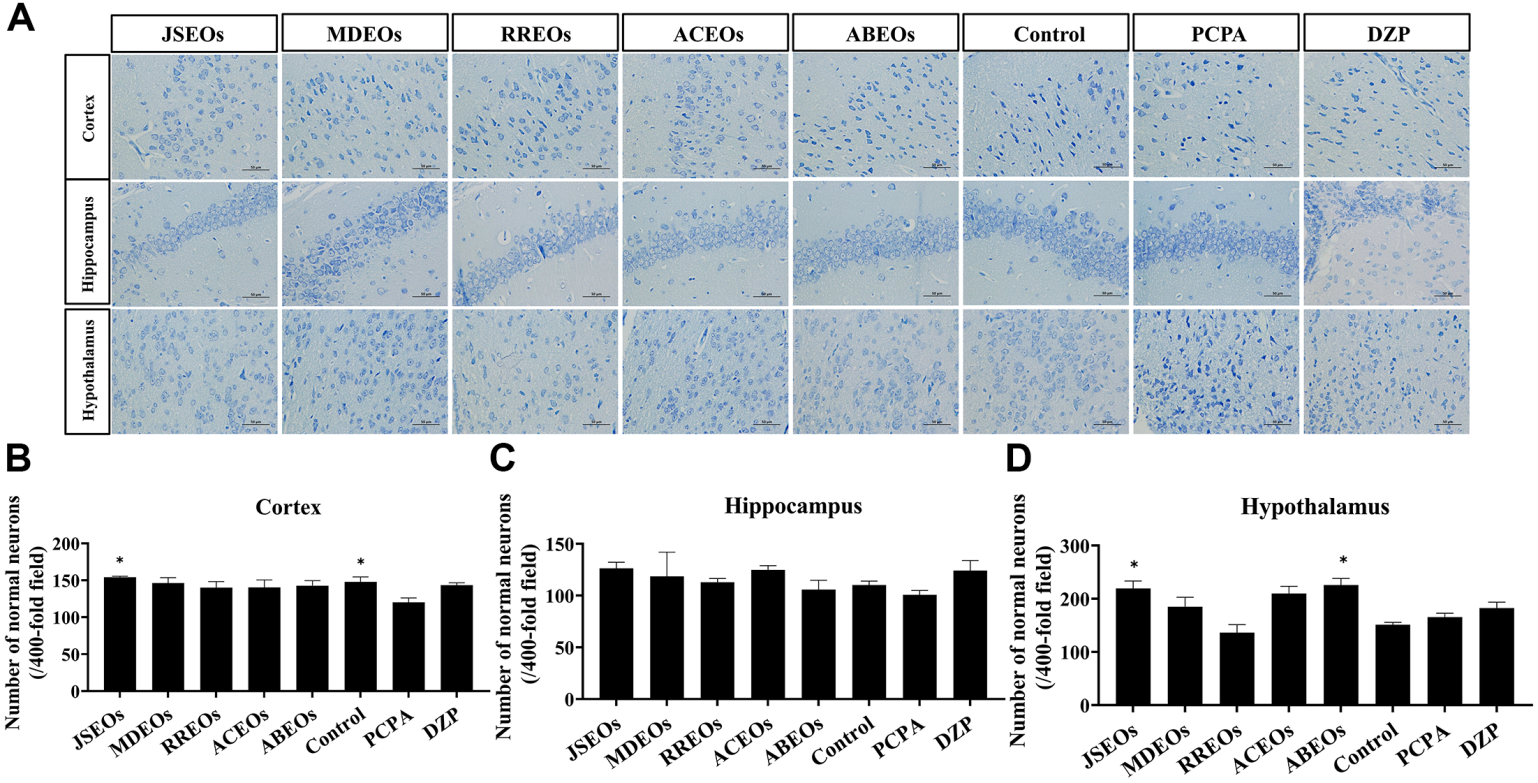
Effects of EOs on neuronal damage induced by PCPA. Nissl staining (A) and normal neuronal counts (B, C, D) in each group; scale bar = 50 μm (x ± s). Significantly differs from the model group, *p < 0.05. EOs, essential oils; PCPA, parachlorophenylalanine. Ordinary one-way ANOVA analysis is used, and the post-hoc tests method is Dunnett's Test.

To clarify whether EOs can reduce neuronal cell death, we used Nissl staining to identify surviving neurons. Nissl staining (Fig. 2A) intact cell morphology. Compared with the PCPA group, the EO-treated groups exhibited well-formed, neatly arranged neurons, suggesting that EOs could effectively improve neuronal damage (Fig. 2A). In particular, the JSEOs groups showed positive neuronal repair effects in the cerebral cortex and hypothalamus (*p* < 0.05), while the PCPA-treated group presented a marked reduction in the number of neurons, disordered cell arrangements, and shrunken cell bodies in the hypothalamus (Fig. 2B and 2D). Compared with PCPA group, ABEOs showed significant neuronal protection in the hypothalamus (*p* < 0.05) (Fig. 2D). In the hippocampus, the therapeutic effects of JSEOs and ACEOs were comparable. Although all EOs had a certain reversal effect on neuronal damage, it did not show significant effect (Fig. 2C).

### Immunohistochemistry Analysis

Given that 5-HT_1A_R and GABA_A_Rα1 are abundantly expressed in the cortex, hippocampus, and hypothalamus, neurogenesis in these areas is regulated by fear-related behavior. To determine whether 5-HT_1A_R and GABA_A_Rα1 were associated with EO-mediated sedative and hypnotic effects, changes in protein and mRNA levels of 5-HT_1A_R and GABA_A_Rα1 in the cortex, hippocampus, and hypothalamus were examined using immunohistochemistry, RT-qPCR, and western blotting.

**Fig. 3:**
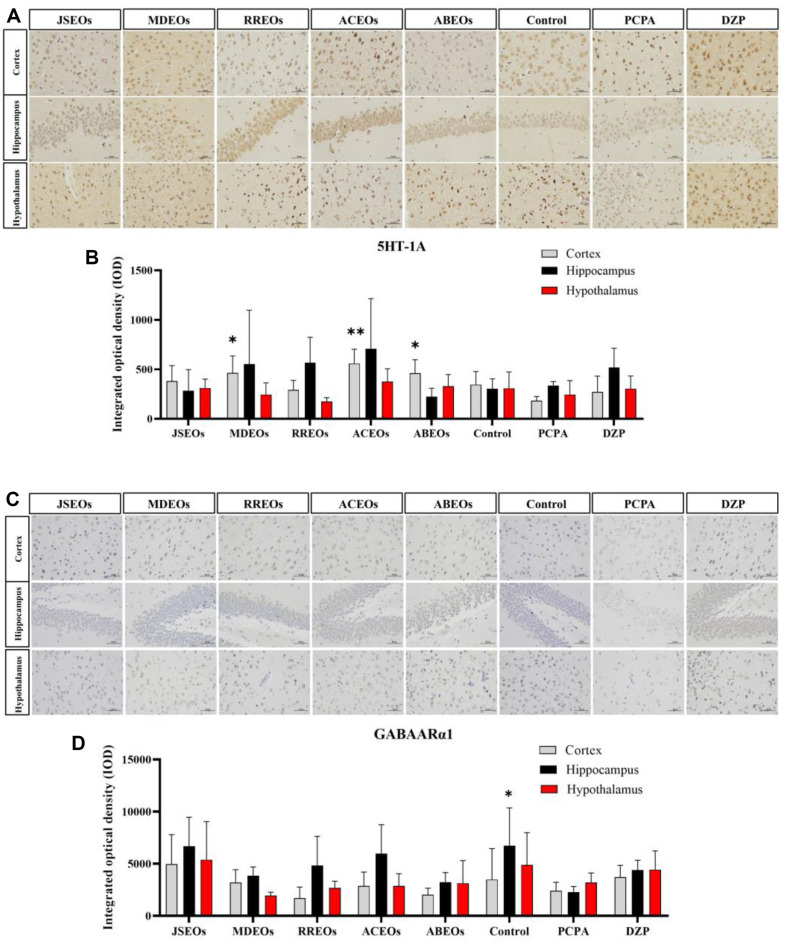
Effects of EOs on expression levels of 5HT_1A_ and GABA_A_Rα1 in brain tissues determined by immunohistochemistry. (**A, B**) Representative image of 5HT_1A_ immunostaining in three brain areas and densitometric analysis of 5HT_1A_ expression (x ± s). (**C, D**) Representative image of GABA_A_Rα1 immunostaining in the three brain regions and densitometric analysis of GABA_A_Rα1 expression. (x ± s). Scale bar = 50 μm. Significantly differs from the model group, *p < 0.05 and **p < 0.01. EOs, essential oils. Ordinary one-way ANOVA analysis is used, and the post-hoc tests method is Dunnett's Test.

As determined by immunohistochemistry, we observed a significant increase in the expression level of 5-HT_1A_R in the samples treated with MDEOs, ACEOs, and ABEOs, manifested as dark brown staining (Fig. 3A). The *M. denudata* EO (MDEO)-treated, *A. citriodora* EO (ACEO)- treated, and *A. balsamea* EO (ABEO)-treated groups exhibited increased expression of 5HT_1A_ when compared with the control group. Compared with the control group, these three groups exhibited markedly increased 5HT_1A_ expression in the cortex (*p* < 0.05)(Fig. 3B). Although JSEO-treated and RREO-treated groups showed increased 5HT_1A_ expression in different examined brain regions, the differences were insignificant. We detected no significant difference between hippocampus and hypothalamus expression levels. Similar to 5HT_1A_, immunohistochemistry indicated that the GABA_A_Rα1 expression was enhanced in EO-treated mice (Fig. 3C). The cortical region exhibited higher levels of GABA_A_Rα1 in response to JSEOs, MDEOs, and ACEOs compared to the PCPA group. Within the hippocampal region, all EOs treatments resulted in an elevation of GABA_A_Rα1 levels relative to the PCPA control. Across the three brain regions examined, JSEOs demonstrated the most pronounced therapeutic effects, although no statistically significant differences were detected among the treatments (Fig. 3D).

### Western Blot Analysis

**Fig. 4:**
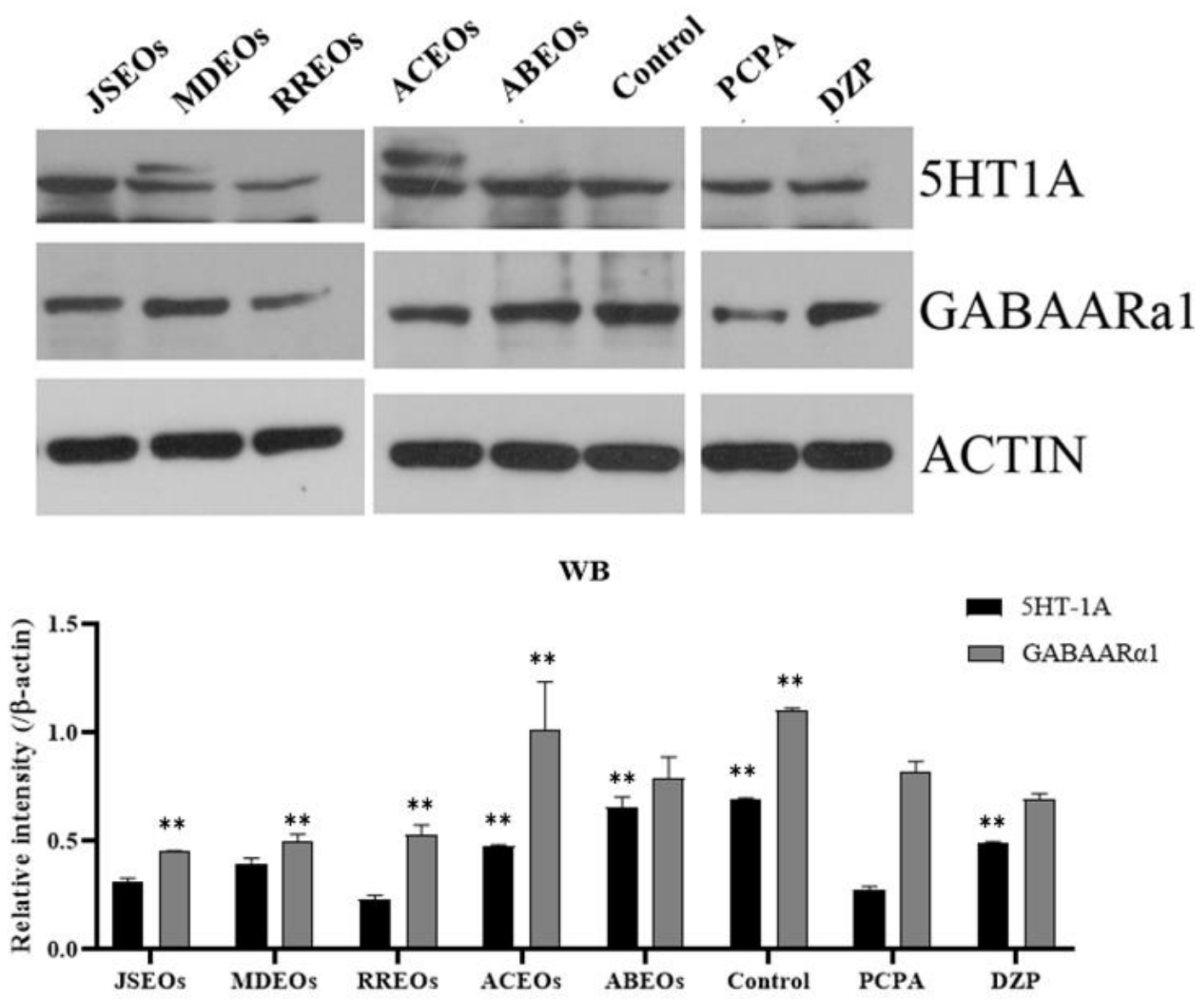
Effect of EOs on 5HT_1A_ and GABA_A_Rα1 levels detected by western blotting in brain homogenates after PCPA treatment in mice. The 5HT_1A_ and GABA_A_Rα1 protein levels in different groups were expressed as a ratio to that of the corresponding β-actin in brain homogenates. Significant differences from the model group, *p < 0.05. Values are expressed as mean ± SEM (*n* = 10). EOs, essential oils; PCPA, parachlorophenylalanine.

As shown in Fig. 4, the expressions of 5-HT1A and GABA_A_Rα1 in control group and ACEOs were significantly higher than those in the PCPA group (*p* < 0.01). At the same time, the expression of 5HT_1A_ in the ABEOs group was significantly higher than that in the PCPA group (*p* < 0.01), and the expression of 5HT_1A_ in MDEOs was also increased. However, the expression of GABA_A_Rα1 in JSEOs, MDEOs, and RREOs was significantly reduced. Actin was used as a loading control for the immunoblotting experiments. These results indicated that EOs may regulate sedation and hypnosis by modulating changes in protein and mRNA levels associated with the 5-HT1AR and GABA_A_Rα1 systems, affecting respective receptors in the mouse brain.

### Cytotoxicity of Five EOs

**Fig. 5:**
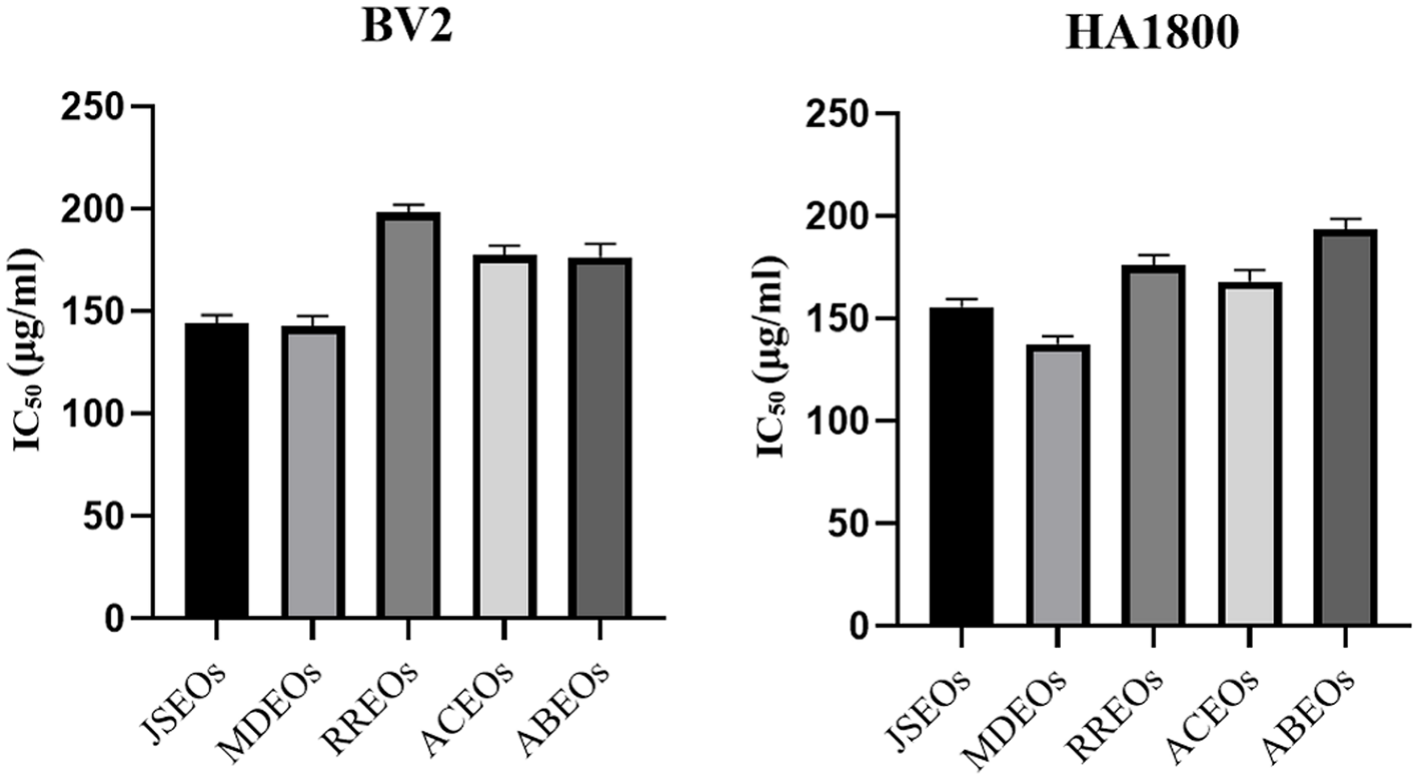
Concentration required to halve the number of surviving cells in each group of essential oils.

The results of the cytotoxic MTT assay were shown in Fig. 5. MDEOs had the lowest IC_50_ value and RREOs had the highest IC_50_ value for BV2 cells, indicating that MDEOs was more toxic to BV2 cells than the other treatments, while RREOs had the lowest toxicity. For HA1800 cells, MDEOs had the lowest IC_50_ value, while ABEOs had the highest IC_50_ value, indicating that HA1800 cells were relatively less tolerant to MDEOs and more tolerant to ABEOs.

### Inflammatory Cytokine Secretion in BV2 Cells

**Fig. 6:**
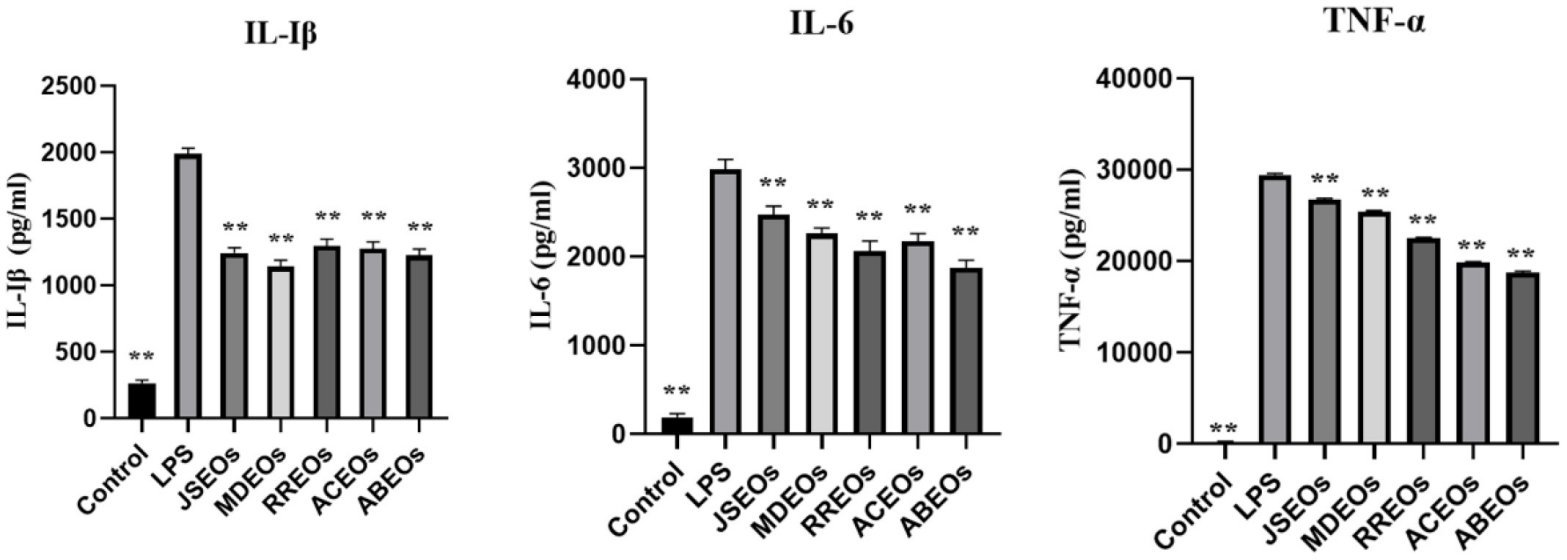
IL-6, IL-1β, and TNF-α levels, as detected by enzyme-linked immunosorbent assay (ELISA). *p < 0.05, **p < 0.01 vs LPS group.

As the typical pro-inflammatory cytokines, IL-6, IL-1β, and TNF-α play important roles in the inflammatory response. ELISA was used to determine the levels of IL-6, IL-1β, and TNF-α in the culture supernatant of BV2 cells, and the results were shown in Fig. 6. The levels of the three pro-inflammatory cytokines in the LPS group were significantly higher than those in the control group. The levels of IL-6, IL-1β, and TNF-α were significantly reduced after treatment with five EOs, indicating that the five EOs inhibited the secretion of pro-inflammatory cytokines in LPS-activated BV2 cells.

### RT-qPCR Analysis

**Fig. 7:**
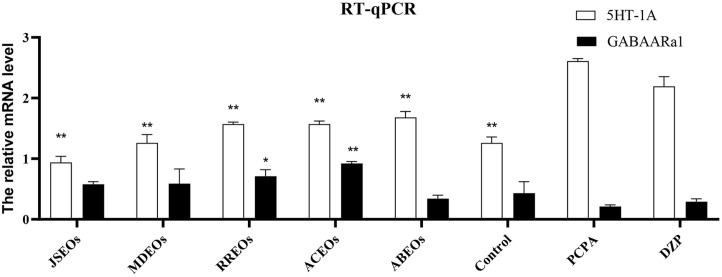
Relative expression of mouse 5HT_1A_ and GABA_A_Rα1 protein genes determined by RT-qPCR. Significant differences from the model group, *p < 0.05 and **p < 0.01 vs PCPA group. Values are expressed as mean ± SEM (*n* = 10). RT-qPCR, reverse transcription-quantitative PCR.

As shown in Fig. 7, the PCPA- and DZP-treated groups showed a marked increase in 5HT_1A_ expression and a decrease in GABA_A_Rα1 expression when compared with that in the control group. Except for the JSEO-treated group, the mRNA expression of 5HT_1A_ mRNA was elevated in EO-treated groups when compared with that in the control group. Interestingly, the expression of GABA_A_Rα1 mRNA was similar to that of 5HT_1A_, except in the JSEO-and ABEO-treated groups.

## Conclusion

Currently, patients with insomnia often receive non-drug treatment options. Aromatherapy is considered a superior strategy to improve sleep quality in people with insomnia [25]. Aromatherapy uses a pure plant essence, which has no drug side effects and is not addictive. In addition, it enters the body easily, and its use is simple and convenient. The effectiveness of aromatherapy has been widely confirmed, for example, psychological rehabilitation of American army members, treatment in children with autism, and strategy to improve the immunity of children with AIDS. Aromatherapy has been widely developed in Chile and Peru [26, 27]. In ancient times, people used natural plants to achieve health care and cure diseases. Gradually, these methods have evolved into the currently available aromatherapy. EOs contain more than 100 ingredients, and their chemical composition determines their therapeutic properties. EOs act through the skin, channels, and collaterals to the nervous, hormone, circulatory, and immune systems to relieve the body and mind, enhance conditioning and metabolism, and promote physical health and psychological pleasure [28]. These findings provide new avenues for treating several central nervous system diseases.

The primary objective of the present study was to determine the hypnotic effects of aromatherapy with five EOs, and the association between insomnia and the expression of related proteins in brain tissues. Linalool, an important regulatory substance, can regulate the expression of 5-HT in the brain by inhibiting neurotransmitter abnormalities and neuronal apoptosis targets, ultimately increasing the expression of GABA_A_Rα1, promoting the binding of GABA and GABA_A_ receptors, and enhancing the inhibitory function of GABAergic nerves to achieve sleep. Studies have shown that linalool can enhance GABA energy current in vitro in allosteric manner. Specifically, only oxidized linalool metabolites at C-8 have a positive effect on GABA energy current, while hydroxylated or carboxylated derivatives at C-8 have no effect [21]. This finding suggests that linalool affects its regulatory efficacy on GABA_A_ receptors through specific metabolic modifications, which is an indirect mechanism of action. In one study, Harada *et al*., linalool odor reduced anxious behavior in mice, and this effect disappeared in mice with destroyed olfactory neurons, suggesting that linalool affects the 5-HT1A receptor through olfactory signaling [29]. In recent years, modulation of the cholinergic system has afforded improvements in several brain and neurological diseases [30]. These reports have confirmed the presence of active components, such as linalool, β-Ocimene, caryophyllene oxide, and δ-Cadinene, in these five EOs, potentially associated with altered expression of 5-HT and GABA_A_Rα1 protein levels.

In summary, we investigated the major components and antidepressant pathologies of volatile oils derived from *J. sambac*, *M. denudata*, *R. rugosa*, *A. citriodora* and *A. balsamea*, which are commonly used in aromatherapy and related health supplements and fragrances. After identification, citronellol (23.78%) is the main component of RREOs, linalool (68.84%) of MDEOs, α-Farnesene (28.42%) of JSEOs, linalool (60.27%) of ACEOs and acetic acid bornyl ester (38.85%) of ABEOs. Studies have shown that citronellol can improve insomnia symptoms by upregulating 5HT_1A_ receptor and GABA_A_. Linalool also shows active sedative and anticonvulsant central activities. It has also been demonstrated that acetic acid bornyl ester and α-Farnesene exhibit the anti-inflammatory and antioxidant activity.

As MTT assay results suggest, some essential oils may have cytotoxic effects. Notably, one study showed that high doses of *A. citriodora* essential oil after percutaneous absorption in vitro had significant effects on human skin cell function, including loss of integrity and solubility of the corneum, cell necrosis, and cellular vacuolation [31]. Therefore, more in-depth analysis of the dose and toxicity of these essential oils is necessary before they are used in human therapy.

Our data were generated using the PCPA-induced insomnia model in KM mice. We examined the effects of different EO treatments on neurotransmitter function and synaptic inhibition in insomnia mice and harvested tissues. Herein, we found that oils from all five plants significantly increased body weight, improved sleep quality, and upregulated related protein expression in the brain tissue of mice. It has been speculated that EOs may influence appetite by regulating the activity of neuropeptides in the hypothalamic arch nucleus (ARC) that promote appetite (orexigenic) and suppress appetite (anorexigenic) [32]. Although our study did not directly measure appetite, it can be believed that the weight change is due to EOs regulating appetite. Among these, ACEOs afforded superior effects for insomnia treatment. The plant EO component may enter the brain tissue by crossing the blood-brain barrier, subsequently inhibiting the release and transmission of audit warming activity in the hippocampus and amygdala of the limbic system, promoting the binding of GABA to GABA_A_ receptors, increasing the frequency of Cl- channel opening and cell membrane hyperpolarization, enhancing the inhibitory function of GABAergic nerves, and preventing neuronal apoptosis in the brain [22, 33]. In addition, the five EOs could induce changes in the humoral environment to promote regulation of choline receptor levels, secretion of 5HT_1A_, and its response to activation of the intracellular second messenger cascade. The pharmacological mechanisms of sleep warrant further elucidation using metabolomics. Multiple factors may influence the pathogenesis of insomnia. The results of this study suggest that essential oil components may have a direct effect on sleep regulation pathways, but the specific mechanisms of action of these components in the brain need to be further clarified. Therefore, we recommend that future studies should include in vivo imaging and pharmacokinetic studies to determine the BBB penetration of these essential oil components, which is essential to fully understand their therapeutic potential.

Our study found that in the JSEOs and ABEOs treatment groups, we observed significant neuronal repair in the cerebral cortex and hypothalamus regions, showing more complete and neatly arranged neurons. This protective effect may improve sleep quality by reducing neuronal damage and maintaining neuronal structural integrity. The number of neurons in the essential oil treated group did not decrease sharply, which may indicate that essential oils help prevent neuronal apoptosis. Apoptosis is a programmed cell death process that may play a role in neurodegenerative diseases and sleep disorders. By reducing apoptosis, essential oils may help maintain the number of neurons, which can have a positive impact on sleep quality.

Aromatherapy can be regarded as safe sleep therapy. To understand the application of insomnia in traditional Chinese medicine therapy and study its pathogenesis. The natural and main ingredients of other sleeping pills have been systematically examined. The present study investigated underlying mechanisms through which choline levels are enhanced by different natural plant EOs. Based on our findings, we clarified the use and dosage of aromatherapy to meet the growing demand for natural functional healthcare products for aromatherapy product development and research based on theoretical foundations.

## Figures and Tables

**Table 1:** The Latin name, local name, extraction site, voucher specimen number, collection time, and storage location of five plants.

Latin name	Local name	Extraction site	Voucher number	Collection time	Storage location
*Jasminum sambac*	Moli	Flowers	2018-J001	2018.08	School of Biomedical and Pharmaceutical Sciences, Guangdong University of Technology
*Magnolia denudata*	Mulan	Flowers	2018-M002	2018.08	
*Rosa rugosa*	Meigui	Flowers	2018-R003	2018.08	
*Aloysia citriodora*	Ningmengmabiancao	Whole	2018-A004	2018.08	
*Abies balsamea*	Jiaolengshan	Leaves	2018-A005	2018.08	

**Table 2:** Primer sequence for RT-qPCR.

Gene	Sequence of primer (5’→3’)		Annealing temperature (°C)
	Forward primer	Reverse Primer	
5HT_1A_	CCAACTATCTCATCGGCTCCTT	CTGACCCAGAGTCCACTTGTTG	60
GABA_A_Rα1	ATGACAGTGCTCCGGCTAAAC	AGTGCATTGGGCATTCAGCT	60

**Table 3:** Retention index (*RI*) and relative content (%) of each compound identified from essential oils.

No.	Compounds ^i^	RT (min) ^ii^	RI ^iii^	Exp. RI ^iv^	Ref.	Molecular formula	Classification	Relative content (%)				
								RREOs ^v^	MDEOs	JSEOs	ACEOs	ABEOs
1	β-Ocimene	3.03	861	-	2	C_2_H_16_	Monoterpene hydrocarbon	-	2.32	-	1	-
2	Terpinolene	3.58	916	978	3	C_2_H_16_	Monoterpene hydrocarbon	-	-	-	2	3.66
3	cis-3-Hexenyl benzoate	3.65	916	-	-	C_2_H_16_O_2_	Other	-	-	15.1	3	-
4	Linalool	3.76	982	1011	3	C_2_H_18_O	Oxygenated monoterpene	0.13	68.84	16.52	4	60.27
5	(+)-Limonene	4.47	988	1031	1	C_2_H_16_	Monoterpene hydrocarbon	-	-	-	5	-
6	(-)-Citronellene	4.64	1003	-	-	C_2_H_18_	Monoterpene hydrocarbon	3.86	-	-	6	-
7	cis-3-Hexenyl Butyrate	5.2	1046	-	-	C_2_H_18_O_2_	Oxygenated monoterpene	-	-	1.97	7	-
8	Borneol	5.21	1089	1146	7	C_2_H_18_O	Oxygenated monoterpene	-	1.47	-	8	-
9	cis-p-Mentha-2,8-dien-1-ol	7.19	1116	1138	6	C_2_H_16_O	Oxygenated monoterpene	1.98	-	-	9	-
10	Citronellol	8.42	1140	1126	11	C_2_H_20_O	Oxygenated monoterpene	23.78	-	-	10	-
11	Myrtenol	6.24	1143	1191	5	C_2_H_16_O	Oxygenated monoterpene	-	-	-	11	-
12	α-Terpineol	5.72	1155	1189	5	C_2_H_18_O	Oxygenated monoterpene	-	-	-	12	-
13	Safrole	9.59	1178	1257	4	C_2_H_10_O_2_	Oxygenated monoterpene	-	-	-	13	-
14	3,7-Dimethyl-2,6-octadien-1-ol	9.26	1178	1210	9	C_2_H_18_O	Oxygenated monoterpene	10.76	-	-	14	-
15	Citronellol acetate	12.52	1201	-	-	C_2_H_22_O_2_	Other	6.52	-	0.65	15	-
16	Germacrene B	13.58	1228	-	-	C_2_H_24_	Sesquiterpene hydrocarbon	-	-	0.64	16	3.85
17	Acetic acid bornyl ester	13.98	1238	-	-	C_2_H_20_O_2_	Other	-	-	-	17	-
18	Terpinyl acetate	12.03	1265	1328	6	C_2_H_20_O_2_	Other	-	-	-	18	-
19	α-Caryophyllene	15.92	1287	-	-	C_2_H_24_	Sesquiterpene hydrocarbon	-	3.29	-	19	-
20	δ-Cadinene	16.22	1294	-	-	C_2_H_24_	Sesquiterpene hydrocarbon	1.97	2.08	1.26	20	2.23
21	Phenethyl isovalerate	17.46	1326	-	-	C_2_H_18_O_2_	Other	-	2.19		21	-
22	2-Tridecanone	17.6	1384	1477	1	C_2_H_26_O	Other	6.24	-	-	22	-
23	β-Bisabolene	14.47	1394	1390	3	C_2_H_24_	Sesquiterpene hydrocarbon	-	-	-	23	-
24	Muurolene	17.62	1401	1469	10	C_2_H_24_	Sesquiterpene hydrocarbon	1.96	-	3.45	24	-
25	β-Farnesene	17.78	1437	1458	8	C_2_H_24_	Sesquiterpene hydrocarbon	-	-	0.75	25	-
26	α-Farnesene	17.93	1439	1495	2	C_2_H_24_	Sesquiterpene hydrocarbon	7.87	-	28.42	26	-
27	Nerolidol	24.9	1514	1562	12	C_2_H_26_O	Oxygenated sesquiterpene	-	-	1.45	27	4.7
28	Viridiflorol	19.46	1516	1592	1	C_2_H_26_O	Oxygenated sesquiterpene	-	-	0.91	28	-
29	Caryophyllene oxide	19.84	1542	1582	1	C_2_H_24_O	Oxygenated sesquiterpene	1.09	0.88	0.53	29	-
30	α-Cadinol	22.11	1582	1653	1	C_2_H_26_O	Oxygenated sesquiterpene	-	0.75	2.05	30	-
Total identified/%								74.61	81.82	73.7	74.91	92.18
Monoterpene hydrocarbons/%								14.62	2.32	0	3.66	44.4
Oxygenated monoterpene/%								25.89	70.31	18.49	60.27	4.79
Sesquiterpene hydrocarbon/%								11.8	7.56	35.97	6.28	1.95
Oxygenated sesquiterpene/%								1.09	1.63	3.49	4.7	3.1

^i^Methyl silicon capillary column (30 m x 0.25 mm, 0.25-micron film thickness) for elution sequence of the listed compounds.

^ii^Retention time (RT).

^iii^Retention Index (RI) of N-alkanes (C_6_-C_40_) on the Same Methyl Silicon Capillary Column.

^iV^Literature index. ^1^ Adams, 2007; ^2^ Adams, González Elizondo, *et al*., 2006; ^3^ Ai, Yong, *et al*., 2023; ^4^ Khan, Srivastava, *et al*., 2003; ^5^ Adams, González Elizondo, *et al*., 2006; ^6^ Marongiu, Porcedda, *et al*., 2006; ^7^ Juliani, Koroch, *et al*., 2002; ^8^ Shatar S., 2005; ^9^ Allegrone, Belliardo, *et al*., 2006; ^10^ Binder, Turner, *et al*., 1990; ^11^ Khan, Srivastava, *et al*., 2003; ^12^ Schwob, Bessiere, *et al*., 2004.

^V^JSEOs: *Jasminum sambac* essential oils; MDEOs: *Magnolia denudata* essential oils; RREOs: *Rosa rugosa* essential oil; ACEOs: *Aloysia citriodora* essential oils; ABEOs: *Abies balsamea* essential oils.
